# ViSEAGO: a Bioconductor package for clustering biological functions using Gene Ontology and semantic similarity

**DOI:** 10.1186/s13040-019-0204-1

**Published:** 2019-08-06

**Authors:** Aurélien Brionne, Amélie Juanchich, Christelle Hennequet-Antier

**Affiliations:** 0000 0001 2182 6141 grid.12366.30 BOA, INRA, Université de Tours, 37380 Nouzilly, France

**Keywords:** Gene ontology, Functional genomics, Visualization, Cluster analysis, Semantic similarity, Annotation, Enrichment test

## Abstract

The main objective of *ViSEAGO* package is to carry out a data mining of biological functions and establish links between genes involved in the study. We developed *ViSEAGO* in R to facilitate functional Gene Ontology (GO) analysis of complex experimental design with multiple comparisons of interest. It allows to study large-scale datasets together and visualize GO profiles to capture biological knowledge. The acronym stands for three major concepts of the analysis: **Vi**sualization, **S**emantic similarity and **E**nrichment **A**nalysis of **G**ene **O**ntology. It provides access to the last current GO annotations, which are retrieved from one of NCBI EntrezGene, Ensembl or Uniprot databases for several species. Using available R packages and novel developments, *ViSEAGO* extends classical functional GO analysis to focus on functional coherence by aggregating closely related biological themes while studying multiple datasets at once. It provides both a synthetic and detailed view using interactive functionalities respecting the GO graph structure and ensuring functional coherence supplied by semantic similarity. *ViSEAGO* has been successfully applied on several datasets from different species with a variety of biological questions. Results can be easily shared between bioinformaticians and biologists, enhancing reporting capabilities while maintaining reproducibility. ViSEAGO is publicly available on https://bioconductor.org/packages/ViSEAGO .

## Introduction

**Fig. 1:**
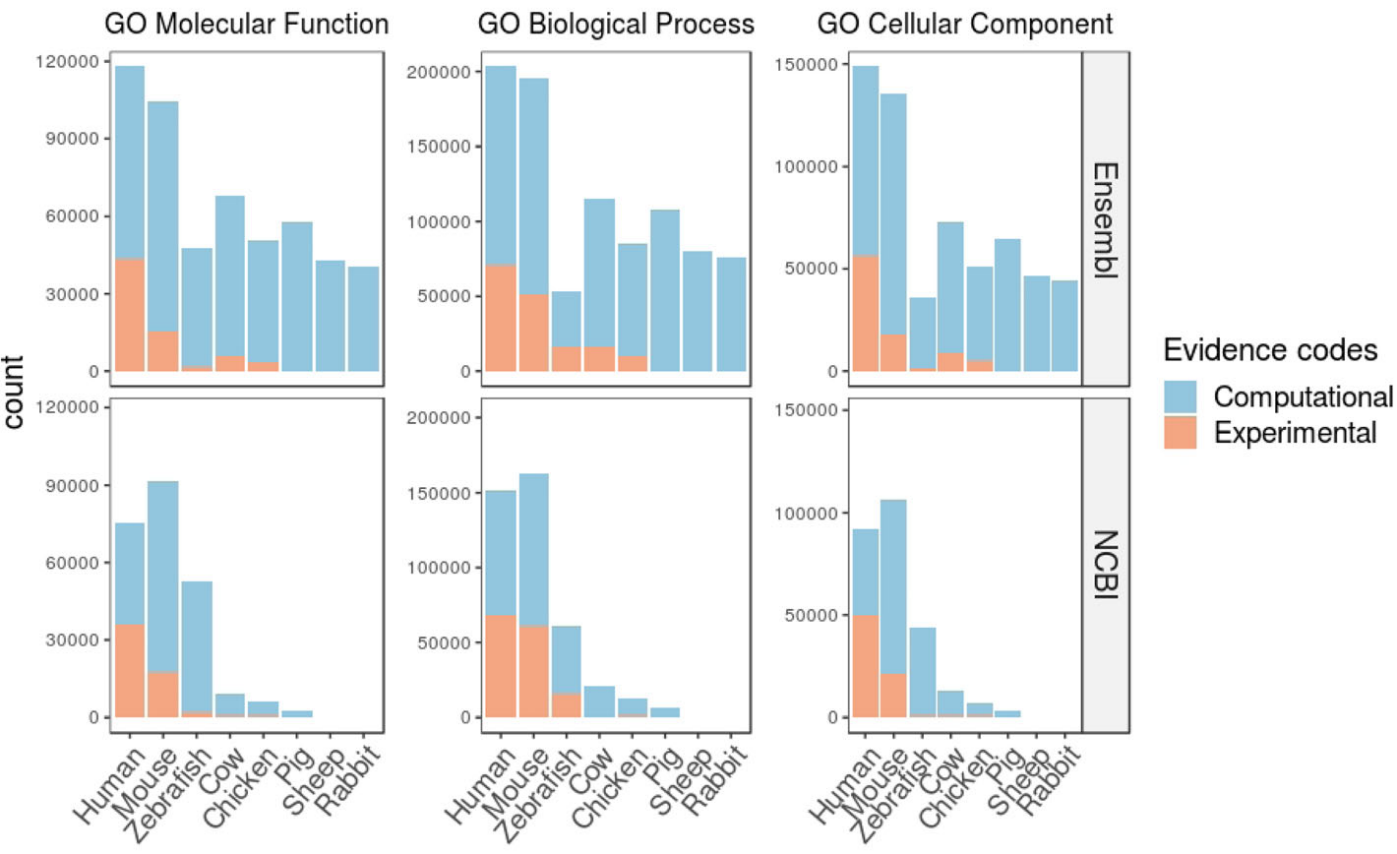
Database impacts on GO annotation. Bar plot of the number of GO annotations available for Molecular Function, Biological Process and Cellular Component category of protein-coding genes in two major databases (NCBI, Ensembl) on three golden standard models with Human, Mouse, Zebrafish and seven livestock animals with Chicken, Cow, Pig, Rabbit, Salmon, Sheep and Trout. Computational (blue) and Experimental (orange) evidence are represented.

Large -omic datasets are nowadays easily produced. While bioinformatical and biostatistical data analyses are quite robust, functional analysis remains a critical step of these high-throughput studies. One essential resource for such analysis is Gene Ontology (GO) [1, 2], that provides an unified vocabulary to describe gene functions (GO terms) and relations between them in three categories: biological processes (BP), molecular functions (MF) and cellular components (CC). GO annotation represents the association between a gene and a GO term. For each category, GO is structured in a graph, where each GO term is a node and edges are relations between GO terms. GO term annotations including GO acyclic graph and GO terms association tables are currently maintained and improved in major databases. However, depending on the database being used, there are important differences between supported species and corresponding genes knowledge. This has a strong impact on GO annotations, enrichment tests and downstream analyses [3]. This statement is clearly illustrated in Fig. 1 with available annotations for three golden standard models such as human, mouse, zebrafish and five livestock species such as cow, chicken, pig, sheep and rabbit. Numbers of GO annotations by category (MF, BP and CC) vary between databases and between species within database for the eight selected vertebrate species. Globally, GO annotations are more inferred computationally for all species, but experimental annotations represent a good part of human and mouse annotations (Fig. 1). Ensembl database contains more annotations than NCBI especially for livestock species due to the use of Ensembl Compara annotation pipeline, which increases the number of terms based on the projection of manually annotated GO terms with experimental evidence type from orthologous genes [4].

**Fig. 2:**
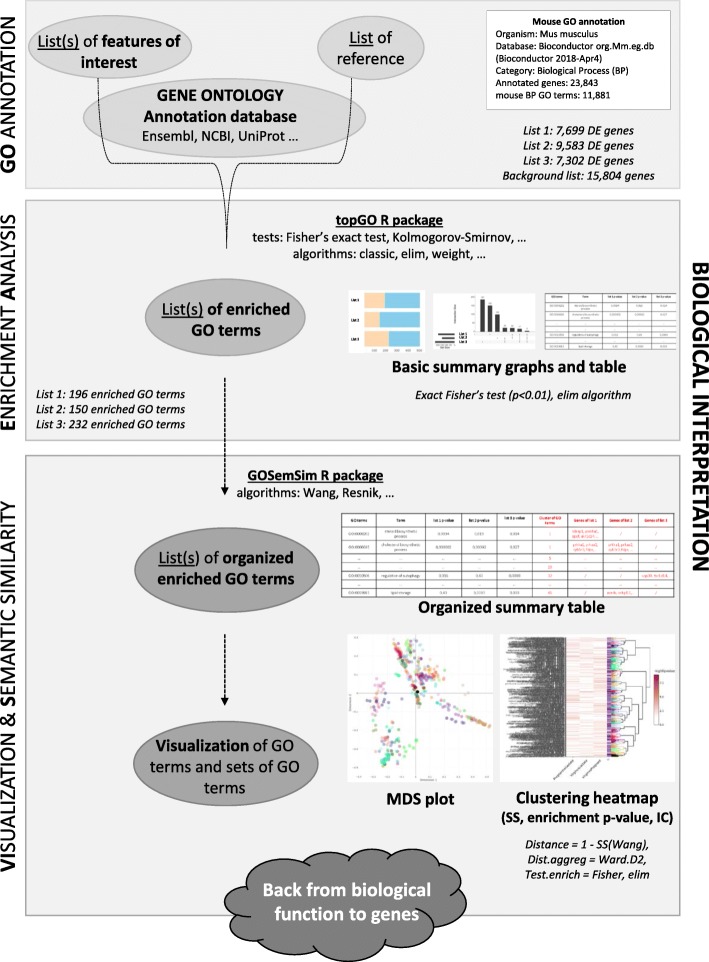
Illustrated ViSEAGO package. A complete ViSEAGO analysis is presented from annotation of lists of features, enrichment tests to organization and viszualisation of GO terms thanks to semantic similarity. In *italic*, illustration of ViSEAGO features using case 1 study

Functional enrichment analysis consists in finding which GO terms are significantly over-represented using GO annotations. Several algorithms and tools for functional enrichment test have been developed [5]. High-throughput studies produce large-scale lists of enriched GO terms, especially in the context of multi-factor experiments. The aim of functional analysis is then to explore lists of GO terms and facilitate biological interpretation. The GO graph provides meaningful links between GO terms, based on the various relationships (*is_a, part_of, related_to, regulates…*). Closest GO terms in the graph share high semantic similarity (SS) and also functional meaning. Indeed, SS is based on the likeness of meaning between biological features. In the last decade, many tools have been developed to compute SS between GO terms and sets of GO terms [6, 7] in different biological applications. SS methods are divided into three categories: those only based on term frequency in a corpus (IC-based, like Resnik’s method [8]), those only based on hierarchical relationships between terms (graph-based like Wang’s method [9]), and those based on hybrid method (like GOGO algorithm [10]). To our knowledge, several tools are implemented to run enrichment tests combined to a downstream analysis that organized the output (using semantic similarity or other algorithms). Surprisingly, only few tools provide support for visualization of lists of GO terms and easier biological interpretation (Table 1) [11–15]. We developed *ViSEAGO* to carry out a data mining of biological functions supported by GO terms and establish links between terms and genes involved in the scientific study. The acronym stands for three major concepts used in the package (Fig. 2): **Vi**sualization, **S**emantic similarity and **E**nrichment **A**nalysis of **G**ene **O**ntology (*ViSEAGO*). By using last current GO annotations, users can at once easily perform multiple enrichment tests on large datasets from complex experimental design. It provides interesting functionalities to organize biological functions into clusters by using GO semantic similarity, an adapted distance computations between GO terms. *ViSEAGO* captures functional similarity based on GO annotations by respecting the topology of GO terms in the GO graph. Hence, it allows enhancing classical functional GO analysis (Table 1). Moreover, through an user-friendly package developed in R language, it facilitates biological interpretation, supported by GO annotations, using several visualizations, like dendrogram of GO terms, MDS of GO terms, heatmap of enrichment *p*-values. It allows data mining of GO terms at different scales, from one term to cluster of GO terms and eventually groups of clusters.

**Table 1:** Description of functionalities supported by different tools focused on biological interpretation from GO annotation

Tool	Interface, Langage	GO Annotation Database	Input identifiers for GO annotation	Enrichment test	GO terms SS	Sets of GO terms SS	Visualization	Multiple lists	Graph interactivity
David https://david.ncifcrf.gov/ [11, 12]	Web	Fixed release, Ensembl, Entrez, Uniprot	GeneID, Ensembl gene ID, Affymetrix probes, Illumina ID, Agilent ID (Do not allow lists of > 3000 identifiers)	Fisher Exact (EASE)	No (used gene’s Kappa similarity)	No	summary table, bar plot, Gene-term 2D view, clustering (centered genes)	Yes	No
ClusterProfiler Bioconductor [13]	R	Current release, Bioconductor databases	GeneID, Ensembl gene ID, can be converted in the module	Hypergeometric	IC-based, Graph-based (computed)	Yes	summary table, bar plot, dot plot, enrichment map, network	Yes	No
gProfiler https://biit.cs.ut.ee/gprofiler/ [14]	R, Web	Fixed release, Ensembl supported species	mixed types of gene identifiers converted to Ensembl gene ID	Hypergeometric	No	No	tree like list of enriched GO terms, summary table	Yes	No
REVIGO http://revigo.irb.hr/ [15]	Web	Fixed release, UniProt and supported several species	No	No	IC-based (computed for visualization)	No	summary table, scatter plot, interactive map, TreeMap, export R plot (centered GO terms)	No	Yes
ViSEAGO Bioconductor	R	Old and current release, Ensembl, Entrez, Uniprot, Bioconductor databases	GeneID, Ensembl gene ID, Uniprot ACC	Fisher Exact	IC-based, Graph-based (computed for visualization)	Yes	summary table, bar plot, upset, MDS plot, clustering (centered GO terms)	Yes	Yes

Based on the topology of the GO graph, thanks to the use of semantic similarity, it allows to look for functional coherence in large dataset and to establish relationships between genes and functions. We demonstrate its use with publicly available RNA-seq datasets (mouse and chicken) and MeDIP dataset (cattle) with different annotation databases.

## Methods

Through *ViSEAGO*, a functional analysis is conducted using the following steps: reading one or multiple lists of genes of interest associated with a reference gene set (i.e. gene background) and loading the last current GO terms’ annotations from selected database in section “**GO** annotation”, performing functional enrichment tests in section “**E**nrichment **A**nalysis”, computing semantic similarity and visualizing clusters of GO terms in section “**Vi**sualization & **S**emantic similarity” (Fig. 2). *ViSEAGO* offers the advantage of performing all analyses in the same statistical environment R, from DE genes identification to the discovery of biological functions of interest. Although the package was developed with differential expression analysis in mind, it can be used with any list of genes, proteins or genomic features.

### Loading experimental data and GO annotations

First, the selected genes of interest, i.e. differentially expressed genes (DE), are divided into one or more lists of genes depending on experimental design. The reference list can be customized. In the case of differential analysis, the reference list will be the list of all expressed genes. We highly recommend to perform functional analysis with all genes of interest (i.e. without threshold on fold change) to maintain a continuum between underlying biological functions. Depending on species and databases, GO term annotations are then performed on these lists of genes with category BP, MF or CC. Available species (animals, plant, fungi, bacteria...) can be displayed from the selected database with *ViSEAGO::available_organisms* method, before fetching the required GO annotations with the *ViSEAGO::annotate* method. Last current GO annotations are extracted at a given time by querying NCBI EntrezGene, Ensembl or Uniprot databases to ensure reliable functional analysis (Fig. 2, Section “GO Annotation”). To re-use data and reproduce analyses, *ViSEAGO* allows the use of older versions of some databases.

### Functional enrichment analysis

*ViSEAGO* addresses the problem of functional analysis in the context of complex experimental designs and large lists of genes of interest. Functional enrichment tests are performed for each list of genes of interest compared to the gene background. No threshold is applied and results are combined together. The most popular test to perform a functional enrichment analysis is the Fisher’s exact test [5]. *P*-values measure the degree of independence between belonging to the GO term and being enriched. They are unadjusted for multiple testing in this exploratory context. *ViSEAGO* offers all statistical tests and algorithms developed in the Bioconductor *topGO* R package [16], taking into account the topology of GO graph by using *ViSEAGO::create_topGOdata* method followed by the *topGO::runTest* method. A table of results that summarizes functional enrichment tests performed for each list of genes is built using *ViSEAGO::merge_enrich_terms* method. The number of enriched GO terms is displayed in a barchart plot using *ViSEAGO::GOcount*. The number of GO terms overlapping between lists of interest is also available in the upset plot with *ViSEAGO::Upset* (Fig. 2, Section “Enrichment Analysis”). Thus, *ViSEAGO* allows comparison of biological functions associated with each list of enriched GO terms in the study. Users can interactively sort the table of results by *p*-values or query by GO term.

### Semantic similarity between GO terms and sets of GO terms

Exploring hundreds of statistically significant GO terms in a “flat” table can be challenging in a complex study with multiple conditions. To catch the group structure from the GO terms data, the choice of measure of similarity between pairs of GO terms is a key criterion. *ViSEAGO* offers therefore several methods based on semantic similarity (SS) to group together enriched GO terms according to their annotation and their topological position in the GO graph. The *ViSEAGO::compute_SS_distance* method is based on the Bioconductor *GOSemSim* R package [17] which implements the five common SS methods between GO terms. Four of the SS algorithms use the information content of a GO term (IC), which is computed as the negative log probability of occurrence of the term in a set of GO terms. A rarely used term contains a greater amount of IC. A Graph-based method as the Wang’s method is also available to compute SS between two GO terms based on the topology of GO graph. The Wang’s method method maintains topology of the GO graph throughout analyses. GO terms are organized into clusters to capture functional coherence in the study before analyzing their enrichment *p*-values.

The *ViSEAGO::compute_SS_distance* method also computes four distance calculations between sets of GO terms [17], including the Best-Match Average (BMA) method which appears to be the best combination approach [18]. BMA calculates the average of all maximum similarities over all pairs of GO terms between two GO term sets, averaged with its reciprocal to obtain a symmetric similarity [18, 19].

### Visualization of clusters of GO terms

*ViSEAGO* helps users to organize GO terms using SS in order to interpret functions involved in the study using multidimensional statistical methods. Multi-Dimensional Scaling (MDS) and clustering heatmap plots are used to compare functional profiles as a whole rather than a set of unrelated GO terms (Fig. 2, Section “Visualization and Semantic similarity”). The level of similarity between enriched GO terms defined by SS is explored using a MDS plot generated with *ViSEAGO::MDSplot* method. To go further, a hierarchical clustering creates clusters of enriched GO terms respecting the GO graph structure with *ViSEAGO::GOterms_heatmap* method. An appropriate dissimilarity between enriched GO terms based on SS and an aggregation criterion for the clustering are chosen to reflect the functional coherence of the analysis. Clusters of enriched GO terms are produced by cutting the dendrogram in a static or dynamic mode developed in *dynamicTreeCut* R package [20]. In addition to the dendrogram of GO terms with their description, a heatmap plot is produced with -log10(*p*-value) from functional enrichment test(s) and IC value. The organization of enriched GO terms into clusters respecting the GO graph topology is entirely supported by the dendrogram and enriched by the results of the functional enrichment tests of the study and completed by the value of IC. This clever combination ensures functional coherence and facilitates biological interpretation. In this way, GO terms within the same cluster share similar biological functions.

To gain further insights, relationships between sets of GO terms can be explored using a similar approach. Similarities between sets of GO terms defined by SS are explored using a MDS plot generated with *ViSEAGO::MDSplot* method. Then, sets of GO terms are organized in a colored dendrogram and grouped into clusters using *ViSEAGO::GOclusters_heatmap* method. In addition to the dendrogram, the sets of GO terms are renamed by their first common GO term ancestor and a heatmap of the number of GO terms in each set is produced.

### Reporting and interactivity

Traceability is ensured by recording major used parameters and results at each step of the analysis. *ViSEAGO* provides interesting functionalities to explore the table of results according to *p*-values from enrichment tests and semantic similarity through GO clusters. Moreover, interactive functionalities implemented thanks to *plotly* R package [21] allow a visualization of biological themes at different scales.

## Results

*ViSEAGO* package has been applied to three biological cases to illustrate its functionalities. All functions and specific parameters used for the three cases are specified in additional file 1.

## Case 1: role of alveolar luminal cells in the mouse mammary gland during pregnancy

**Fig. 3:**
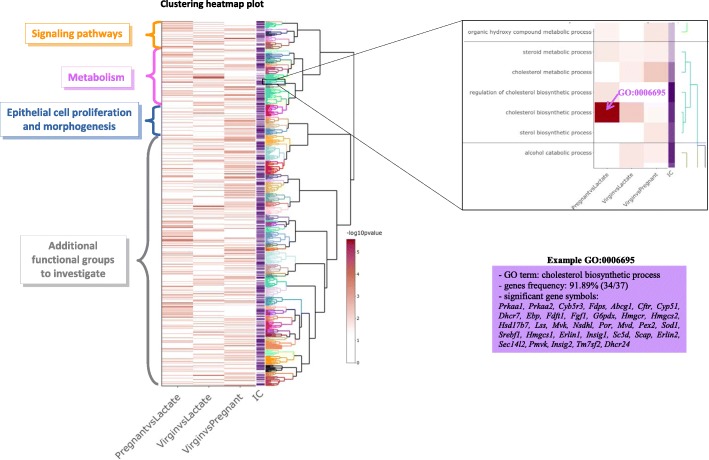
Visualization of ViSEAGO’s functional analysis from mouse RNA-seq with three different transcriptomic datasets. Clustering heatmap plot that combines a dendrogram based on Wang’s semantic similarity distance and *ward.D2* aggregation criterion, a heatmap of -log10(*p*-value) from functional enrichment tests and information content (IC). Focus is made on cholesterol biosynthetic process, a major pathway involved in the study

To illustrate how *ViSEAGO* assesses and compares biological themes, we analyzed the publicly available expression dataset (Gene Expression Omnibus with accession number GSE60450) of luminal cells in the mouse mammary gland [22], using a generalized linear model and quasi-likelihood tests [23], as proposed in edgeR vignette. Among the 15,804 expressed genes, we obtained 7699 DE genes for the comparison pregnant versus lactate, 9583 for the comparison virgin versus lactate and 7302 for the comparison virgin versus pregnant. For each comparison of the study, enrichment of GO BP terms are tested using a Fisher’s exact test with the *elim* algorithm [16] (from *topGO* Package) and mouse GO annotation from EntrezGene database (through Bioconductor org.Mm.eg.db database). In the mouse GO annotation version Bioconductor 2018-Apr4, 23,843 genes are annotated with at least one BP GO term. Enrichment tests revealed respectively 198, 151, and 232 BP enriched GO terms for the three comparisons (*p*-value < 0.01). A clustering heatmap plot (Fig. 3) using SS distance based on *Wang’s* method between enriched GO terms and *ward.D2* aggregation criterion allows to data mine GO terms and capture biological meaning. Enriched GO terms are organized in the dendrogram and branches are colored depending on their cluster assignation. Extracting biological insights from this plot highlights three major pathways: signaling pathways, metabolism, and epithelial cell proliferation and morphogenesis. Those pathways are already discussed in the original publication [8], but ViSEAGO highlighted other interesting groups revealing biological pathways to further investigate. It also pinpoints, using interactive cluster-heatmap zooming capabilities, cholesterol biosynthetic process (GO:0006695) as the most significantly enriched GO term (*p*-value 2.6 10^− 06^ and gene frequency 34/37).

### Case 2: functional genomics of the digestive tract in broilers

**Fig. 4:**
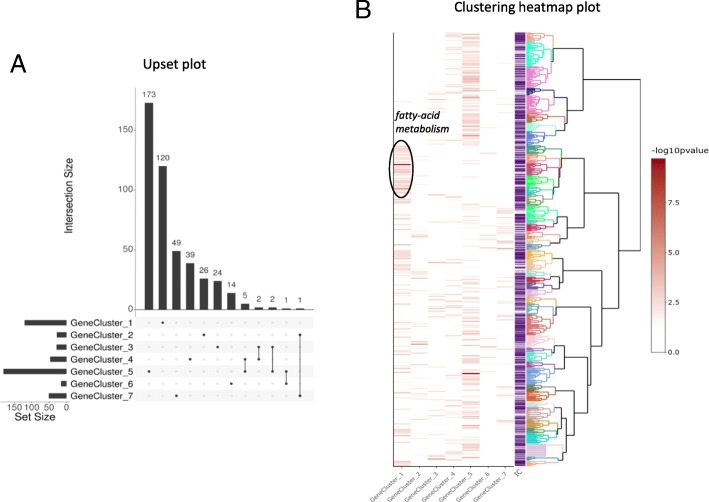
Visualization of ViSEAGO’s functional analysis from chicken RNA-seq with seven different transcriptomic datasets. **a** Upset plot representing overlaps between lists of enriched GO terms, **b** Clustering heatmap plot combining a dendrogram based on Wang’s semantic similarity distance and *ward.D2* aggregation criterion, a heatmap of -log10(p-value) from functional enrichment test(s) of the seven lists of genes and information content (IC)

The aim of the study [24] was to gather knowledge on genes involved in digestive functions in the context of the adaptive capacity of the broilers to changing and even unfavorable dietary conditions. The transcriptome was performed on four digestive tract segments representing the digestive efficiency process (Bioproject accession number PRJNA418230). Differentially expressed genes were identified between the four digestive tract segments by fitting a generalized linear model with edgeR package [23]. Then, DE genes were organized in seven gene clusters to focus on co-regulated genes throughout the digestive tract and their functions. Enrichment tests were performed with *ViSEAGO* package independently for each gene cluster containing at least 300 DE genes up to more than 3000. Annotation by GO for BP was used and Fisher’s exact test with the *elim* algorithm was performed [16]. This algorithm improves the enrichment analysis by taking into account local dependencies between GO terms in GO graph, especially in the case of large data sets. Among the 12,656 expressed genes, 7159 possessed at least one functional GO term in the Ensembl version 94 database (56%). Although functional annotation of the chicken genome remains poor in comparison to model species such as the mouse, functional analysis is still relevant using orthologous relationships with well-annotated species. A total of 456 GO terms were enriched (*p*-value < 0.01) in at least one gene cluster and, among them, 445 were unique (Fig. 4a). This shows that gene clusters are driven by specific functions. The number of enriched GO terms for each gene cluster ranged from 15 to 181, which is mentioned in the output from *ViSEAGO::merge_enrich_terms* method, ensuring traceability of enrichment tests. Results reported in a summary table can be easily and interactively investigated by term (like cholesterol, transport, immune system...) and sorted by *p*-values. Exploring hundreds of statistically significant GO terms in a “flat” table can be challenging in a complex study with multiple conditions. Hence, *ViSEAGO* provides an interactive graphic support to facilitate biological interpretation. Semantic similarity between GO enriched terms are computed using Wang’s SS method to connect together related GO terms by their annotation and position in the GO graph. Enriched GO terms are organized in a dendrogram built from a hierarchical clustering using *ViSEAGO::GOterms_heatmap* based on SS distance between enriched GO terms and *ward.D2* aggregation criterion (Fig. 4b). This interactive visualization allows the user to discover at a glance the general data structure of GO terms and to find out best functions that will explain the digestive efficiency process. For instance, clear groups of organized GO terms related to fatty-acid metabolism are found in the intestine (Fig. 4b). Those functions are essential in the intestine and must work properly for effective digestion.

### Case 3: Hypomethylation in bull sperm targets specific genomic functions

We re-used and re-analyzed Methylated DNA immunoprecipitation (MeDIP) dataset provided by [25] to explore *ViSEAGO*’s functionalities and focus on relationships between sets of GO terms (Gene Expression Omnibus database accession number GSE102960). One aim of the study was to identify hypomethylated CpGs genomic regions and their associated functions from MeDIP datasets in bull sperm in comparison to bovine somatic cells (fibroblasts and liver cells).

Among the 1632 and 3109 hypomethylated regions identified in bull sperm in comparison to fibroblast (FvsS comparison) and liver (LvsS comparison) cells respectively, 732 and 1229 unique genes with a match in regulatory elements (promoter: -1 kb to + 0.1 kb along TSS; downstream: + 1 kb along TES) have been identified using GenomeFeatures R package (https://forgemia.inra.fr/aurelien.brionne/GenomeFeatures).

**Fig. 5:**
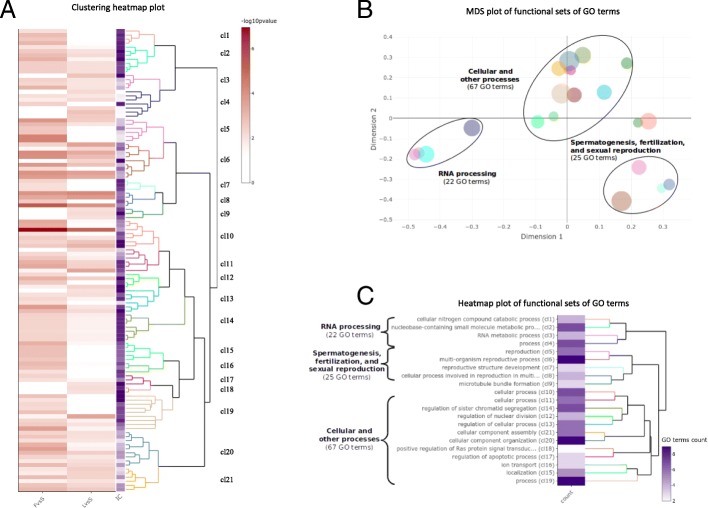
Visualization of ViSEAGO’s functional analysis from cattle with three MeDiP datasets. **a** Clustering heatmap plot combining a dendrogram based on Wang’s semantic similarity distance and *ward.D2* aggregation criterion, a heatmap of -log10(p-value) from functional enrichment tests, and information content (IC). **b** MDS plot based on BMA distance representing the proximities of groups obtained by cutting dendrogram in (**a**). Dot size depends on the number of GO terms within each cluster. **c** Heatmap plot of functional sets of GO terms combining a description of the first common GO ancestor of each set of GO terms, a heatmap with the number of GO terms in each set, a dendrogram based on *BMA* semantic similarity distance and *ward.D2* aggregation criterion

Using Fisher’s exact test, we performed enrichment tests of GO terms associated with BP category and retrieved GO annotation from Ensembl version 81 (version used by the authors). We identified 91 enriched GO terms for FvsS comparison and 53 for LvsS comparison (*p*-value < 0.01). Thirty of the enriched GO terms are shared by the two lists. Several GO terms were already found in the original paper ([25], Fig. 4) and linked to mRNA processing and spermatogenesis (Fig. 5a). For instance, piRNA metabolic process (GO:0034587) was found with an enrichment p-value of 0.026 and is also identified in our study with a p-value of 0.0018 (Fig. 5a, cluster cl3). This enrichment is supported by 4 genes (ASZ1, PLD6, PIWIL2, MAEL) including two out of three already identified (PLD6, MAEL), and reported in the results table (not shown). In complement, we highlighted several GO terms that were not previously found in the study (Fig. 5a) to allow a deeper analysis of the datasets. For instance, the GO term “cellular process involved in reproduction in multicellular organism” (GO:0022412) is the most significant term in both comparisons (1.07 10^− 07^ and 6.76 10^− 06^ for FvsS and LvsS respectively).

A hierarchical clustering using Wang’s SS method and *ward.D2* aggregation criterion that was dynamically cut led to the identification of 21 functional groups of GO terms (Fig. 5a). To easily interpret the biological functions carried out by the 21 groups, SS between the 21 sets of GO terms were computed using BMA distance. Proximities of these functional groups were shown in a MDS plot (Fig. 5b) and a heatmap plot (Fig. 5c). We highlighted three major biological pathways: RNA processing (22 GO terms); spermatogenesis, fertilization, and sexual reproduction (25 GO terms); and cellular and other processes (67 GO terms) notably associated to cellular division (chromosome organization, meiosis and mitosis), signal transduction, RNA transport, and meiosis/spermatogenesis. Part of those GO terms were already found in the original paper but links between them were based on enrichment *p*-values instead of semantic similarity. Using ViSEAGO, sets of GO terms are clearly organized to facilitate functional interpretation taking into account similarities between GO terms and set of GO terms. Thanks to ViSEAGO, we revealed three major functions involved in the study without losing information at the GO term level.

## Conclusions

Functional enrichment analysis remains a major challenge especially on large datasets and complex experimental designs. *ViSEAGO* R package is a generic tool for functional analysis based on Gene Ontology that meets this challenge. The novelty of *ViSEAGO* is providing by the association of the semantic similarity and visualization to focus on biological interpretation with respect for GO graph. *ViSEAGO*’s functionalities are extended compared to most functional analysis tools in three major aspects: (1) emphasize functional coherence by aggregating closely related biological themes based on the GO graph topology; (2) reliability of the functional interpretation using the last current GO annotations; (3) interactive visualization both synthetic and detailed to facilitate biological interpretation. At the end, *ViSEAGO* helps users to perform a reproducible functional analysis and to prioritize genes to investigate.

## Data Availability

Upon publication, *ViSEAGO* R package will be freely available on Bioconductor: https://bioconductor.org/packages/ViSEAGO .

## Abbreviations

BMA: Best-match average
BP: Biological process
CC: Cellular component
DE: Differentially expressed
GO: Gene ontology
IC: Information content
MDS: Multi-Dimensional Scaling
MeDIP: Methylated DNA ImmunoPrecipitation
MF: Molecular function
RNA: RiboNucleic Acid
SS: Semantic similarity
TES: Transcription end site
TSS: Transcription start site
